# Capsule Commentary on Reese et al., Two Randomized Controlled Pilot Trials of Social Forces to Improve Statin Adherence Among Patients with Diabetes

**DOI:** 10.1007/s11606-016-3599-0

**Published:** 2016-02-18

**Authors:** Maria K. Wolters

**Affiliations:** Informatics & Psychology, University of Edinburgh, Edinburgh, UK

At first glance, readers might disagree with the decision to publish a paper that reports not one, but two negative results. However, it is absolutely vital for scientific progress that such findings are shared with the research and the clinical community, because publication bias skews the quality of the evidence.^1^

As this paper reports two carefully designed and executed randomised controlled trials, its lessons are particularly important. In both trials, all participants greatly increased their medication adherence, despite being in a high risk group, older, and of lower socioeconomic status.^2^ All they did was take part in a trial where they received an electronic pill bottle. Additional social feedback, as provided in the intervention arms of the trials, was not needed.

While the authors list a number of possible reasons for this finding, it would have been much better to explore it through a programme of qualitative interviews and focus groups. The data from such qualitative work are key for translating findings from randomised controlled trials into practice.^3^

In this study, the Hawthorne effect—that observation changes behaviour, a phenomenon well-documented in clinical trials^4^—may have been augmented by the fact that all study arms received a special device, the electronic pill bottle. Providing such bottles in itself constitutes an intervention that improves medication adherence.^5^ A well-designed qualitative interview study with a small proportion of the participants in each arm could have helped clarify to what extent those effects might have been present. It would also have provided valuable data on the participants’ motivation for taking part in the study, and highlighted the extent to which the study itself might have been a valuable social force.
